# Physicochemical characterization of polyhydroxybutyrate (PHB) produced by the rare halophile *Brachybacterium paraconglomeratum* MTCC 13074

**DOI:** 10.1186/s12934-024-02324-1

**Published:** 2024-02-22

**Authors:** Teja Mandragutti, Tura Safawo Jarso, Sudhakar Godi, S Sharmila Begum, Beulah K

**Affiliations:** 1 Department of Biotechnology, Andhra University, Visakhapatnam, 530 003 India; 2 Department of Biology (Applied Genetics and Biotechnology Stream), College of Natural Sciences, Salale University, Fiche, Ethiopia; 3 Department of Biotechnology, Dr Lankapalli Bullayya College, Visakhapatnam, 530013 India

**Keywords:** Polyhydroxybutyrate, *Brachybacterium paraconglomeratum*, Physicochemical characterization, Viscosity

## Abstract

**Background:**

Polyhydroxybutyrate is a biopolymer produced by bacteria and archaea under nitrogen-limiting conditions. PHB is an essential polymer in the bioplastic sector because of its biodegradability, eco-friendliness, and adaptability. The characterization of PHB is a multifaceted process for studying the structure and its properties. This entire aspect can assure the long-term viability and performance attributes of the PHB. The characteristics of PHB extracted from the halophile *Brachybacterium paraconglomeratum* were investigated with the objective of making films for application in healthcare.

**Results:**

This was the first characterization study on PHB produced by a rare halophile, *Brachybacterium paraconglomeratum* (MTCC 13074). In this study, the strain produced 2.72 g/l of PHB for.5.1 g/l of biomass under optimal conditions. Methods are described for the determination of the physicochemical properties of PHB. The prominent functional groups CH_3_ and C = O were observed by FT-IR and the actual chemical structure of the PHB was deduced by NMR. GCMS detects the confirmation of four methyl ester derivatives of the extracted PHB in the sample. Mass spectrometry revealed the molecular weight of methyl 3-hydroxybutyric acid (3HB) present in the extract. The air-dried PHB films were exposed to TGA, DSC and a universal testing machine to determine the thermal profile and mechanical stability. Additionally, the essential property of biopolymers like viscosity was also assessed for the extracted PHB.

**Conclusions:**

The current study demonstrated the consistency and quality of *B. paraconglomeratum* PHB. Therefore, *Brachybacterium sps* are also a considerable source of PHB with desired characteristics for industrial production.

## Background

Polyhydroxy alkanoates (PHAs) have been discovered to be a potential and environmentally acceptable alternative to synthetic polymers. Polyhydroxy butyrate (PHB) is a type of PHA polymer that is in high demand due to its biological origin and biodegradability. The mechanical qualities and bioways to produce PHBs make it ideal for replacing polythene. Unlike traditional plastics, PHB can be extensively decomposed by microbes to produce carbon dioxide and water [1]. PHB developed as insoluble inclusions in the cells of a wide range of bacteria that serve as energy granules, which have drawn the interest of researchers in recent years [2]. There have been several findings and facts regarding the natural structure of PHB granules and the physical state of PHB. The native granules contain proteins and lipids that are quickly broken down by cellular depolymerases under favorable conditions [3].

PHB is a member of the polyester family since it contains ester groups in its chemical structure [4]. These polymers can be homopolymers, which contain only one type of 3-hydroxy fatty acid monomer, copolymers which contain two types of 3-hydroxy fatty acid monomers and heteropolymers, which contain 3-hydroxy fatty acids with different chain lengths and other block copolymers, Random PHA copolymers are produced subject to the type of microbe and their growth conditions [5].

The halophiles can survive and even flourish in high saline conditions, while the majority of other species would perish in the same conditions. A few halophilic species of actinomycetes, cyanobacteria and yeast produce PHB, which can be used in place of synthetic polymers in a variety of commercial applications. The occurrence of PHB derivatives in ten different strains of the genus Streptomyces sp. of actinomycetes was investigated. However, only a few studies were available on the utilization of actinobacteria for the PHB synthesis [6]. The extraction and production of PHB at a minimal cost is necessary for emerging into the plastic-dominated industry and this cost reduction could widen the range of applications for biopolymers which will enhance their marketability. Nutrient management, microbe optimization, molecular cloning, co-culture and mixed fermentations are the most acceptable strategies for reducing the cost of production and significantly increasing PHB yield. The application possibilities and production methods of PHB are constantly expanding due to the initiatives in research and development [2].

The Methods such as staining, spectroscopy, chromatography and microscopy are available for screening PHB-producing bacteria and for characterizing PHB [7]. Crotonic acid estimation has long been regarded as a valid method for quantifying PHB. Presently, microbial cytoplasmic PHBs can also be quantified and characterized using advanced scientific methods such as gas chromatography (GC) [8], liquid chromatography(LC) [9], Fourier transform infrared spectroscopy (FTIR) [10, 11] and nuclear magnetic resonance spectroscopy (NMR) [12].

The glass transition temperatures (Tg), melting temperature (Tm) and thermodegradation temperature (Td) of biopolymers are persistently studied to determine the temperature ranges at which the polymers are generated and employed [13]. PHB composites have a crystallinity range of 0 to 70% and can be neither non-crystalline nor very crystalline. Techniques such as FTIR, DSC, and X-ray diffraction could be utilized to determine the crystallinity and compositional analysis [14]. The viscosity of biopolymers is regulated by several parameters, including molecular weight, concentration, temperature, shear rate and structural characteristics. The viscosity of PHB fermented broths and extracts can be determined by the Lovis method [15]. Capillary rheometry, thermal analysis and tensile tests were used to evaluate the strengths of PHB and biodegradable blends [16]. High rheological values tend to increase the processing times of PHB. The characterization of PHB is of paramount importance due to its significant impact on various fields and industries. Characterization ensures uniformity and quality before application development. As a result, examining these behaviors makes it easier to optimize the processing conditions of PHB. The analytical measures discussed above were used to characterize the PHB extracted from the halophile *B. paraconglomeratum*.

## Results

### Bacteria and yield of PHB

The bacteria were isolated selectively and subjected to 16 S rRNA sequencing, deposited in CSIR-IMTECH (MTCC 13074), NCBI (accession: MW899045) and also published [17]. The bacteria produced a substantial quantity of PHB about 2.72 g/L for 5.1 g/L of biomass.

### FTIR analysis

**Fig. 1:**
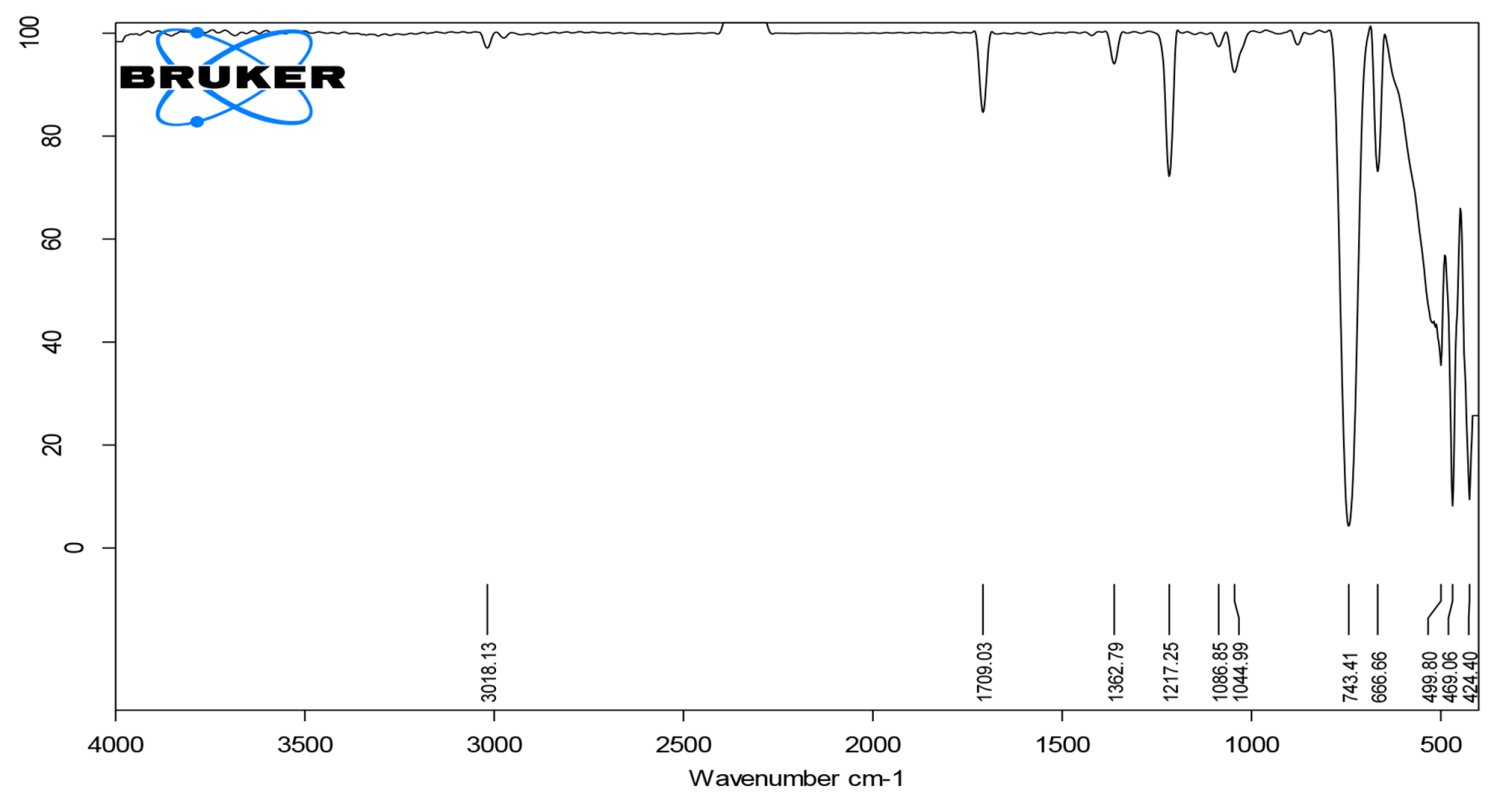
FTIR spectra related to the functional groups of PHB in the extract of *B. paraconglomeratum*

FTIR was used to investigate the functional groups in the molecular structure of the extracted biopolymer. This directly detects the presence of aliphatic C-H bonds, =C-O bonds, =CH bonds and = C-H bonds as well as the deformation and stretching of = O and = C-H bonds. In the extracts of *B. paraconglomeratum*, the carbonyl group was centered at 3019 cm^− 1^_,_ which indicates the presence of longer aliphatic chains. The stretches at 1709 and 1362 cm^− 1^ indicate the C = O, C-H bending or CH_3_. The obtained IR spectra showed typical ester C-O bonds at 1217 cm^− 1^. The strong peak in the 700–800 cm^− 1^ range corresponded to the stretching vibrations of the C-Cl bond of chloroform. The IR spectra (Fig. 1) revealed that the type of biopolymer produced by *B. paraconglomeratum* was most likely to be polyhydroxy butyric acid.

### GCMS analysis

**Fig. 2:**
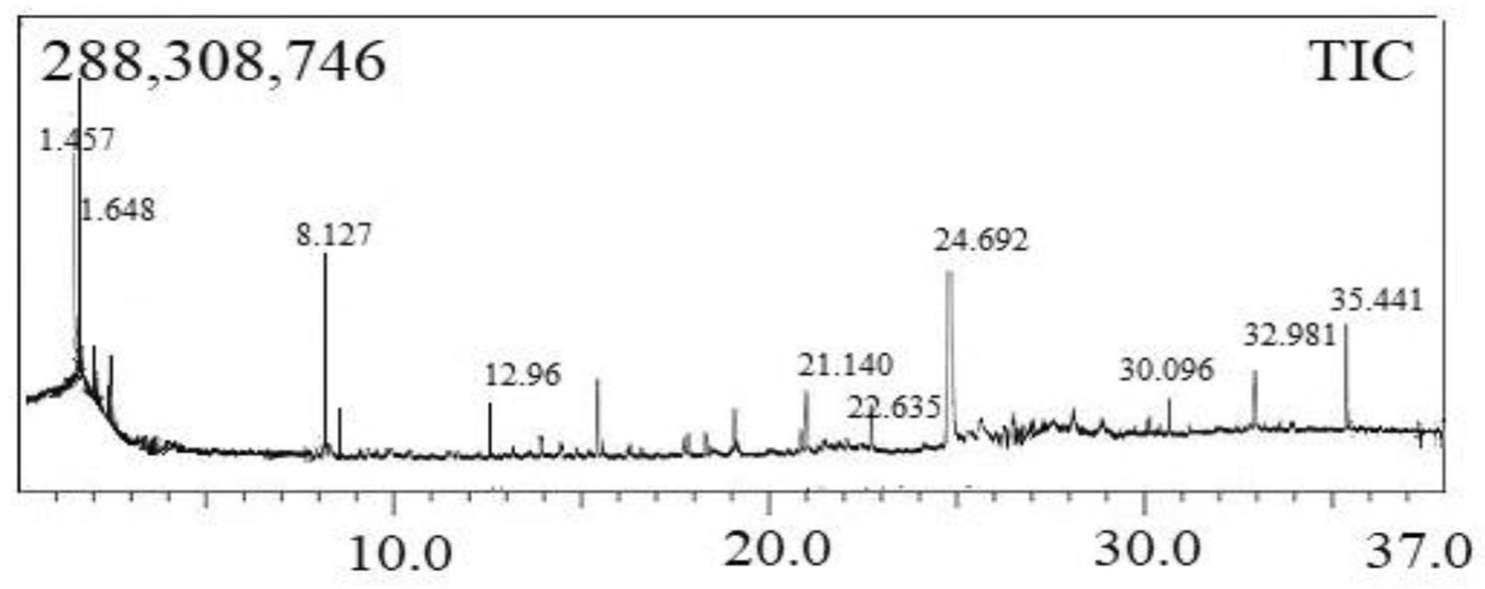
Gas chromatography analysis of derivatives PHB obtained from *B. paraconglomeratum*

The PHB sample was subjected to methanolysis and the methylesters displayed fragmentation patterns in GCMS, allowing to identify the derivatives of PHB produced by *B. paraconglomeratum MTCC 13074*. The outcomes were compared to those of standard results in the PubChem database and sigma reference. The chromatogram (Fig. 2) of the biopolymer extract revealed four major peaks with retention times of 21.140 min, 22.635 min, 30.096 min, and 35.441 min respectively. They are tetradecanoic acid, 13-docosenoic acid methyl ester, (Z)- hexadecanoic acid methyl ester and 9-octadecenoic acid (Z)- 2-hydroxy-3-[(1-oxohexadecyl) oxy] propyl ester. Table 1 shows the principal peaks and supports the presence of polyhydroxy butyric acid (PHB) in *B. paraconglomeratum* extracts. The x-axis in Fig. 2 represents retention time (min), and the y-axis represents signal intensity.

**Table 1:** List of PHB derivatives detected in GCMS

Peak	R. Time	Area	Area%	Height	A/H	Base m/z	Name
5	21.140	15,087,841	0.45	4,477,091	3.37	55.15	Tetradecanoic acid
6	22.635	7,171,994	0.21	3,440,255	2.08	55.05	13-Docosenoic acid, methyl ester, (Z)-
7	30.096	161,531,637	4.81	27,304,955	5.92	43.10	Hexadecanoic acid, methyl ester
9	35.441	378,738,659	11.28	48,450,771	7.82	57.10	9-Octadecenoic acid (Z)-2-hydroxy-3-[(1-oxo hexadecyl)oxy]propyl ester
		3,356,296,179	100.00	1,130,547,317			

### NMR analysis

**Fig. 3:**
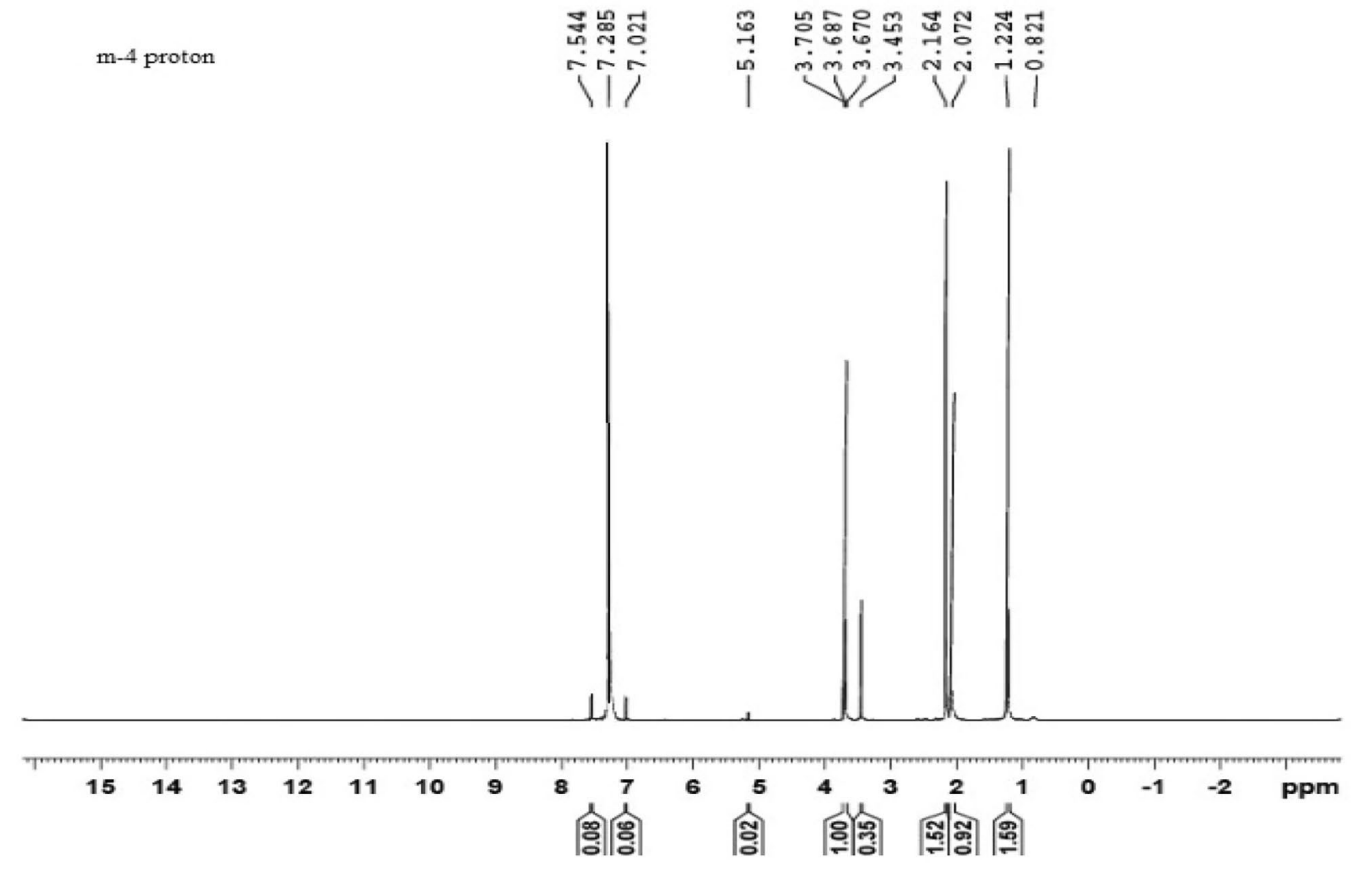
^1^H-NMR spectra of PHB produced by *B. paraconglomeratum*

**Fig. 4:**
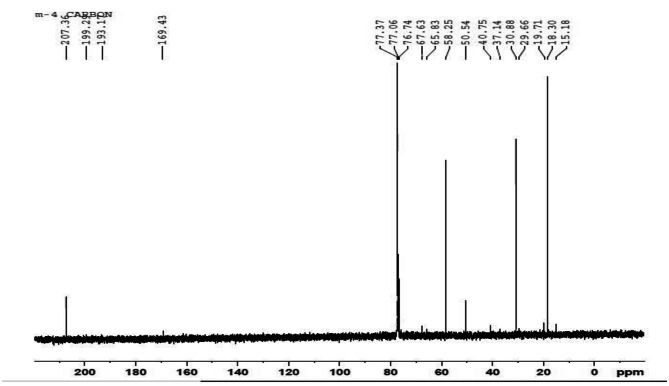
^13^C-NMR spectra of PHB produced by *B.paraconglomeratum*

The ^1^H NMR spectra (Fig. 3) of *B. paraconglomeratum* PHB extracts showed resonances for the side groups of hydroxybutyrate i.e., (-CH3) at 1.224 ppm, a singlet for (-CH-) at 5.163 ppm and a doublet for (CH2) at 2.164, and 2.072 ppm. Correspondingly, the ^13^C NMR spectra (Fig. 4) indicated the peaks at 19.71, 40.75, 67.63 and 169.43 ppm for (CH3), (–CH2–), (–CH–) and (–C–). The resonances of methyl, methylene, methane, ester groups and carbonyl carbon atom confirmed that the polymer obtained was PHB.

### Mass spectrometer analysis

**Fig. 5:**
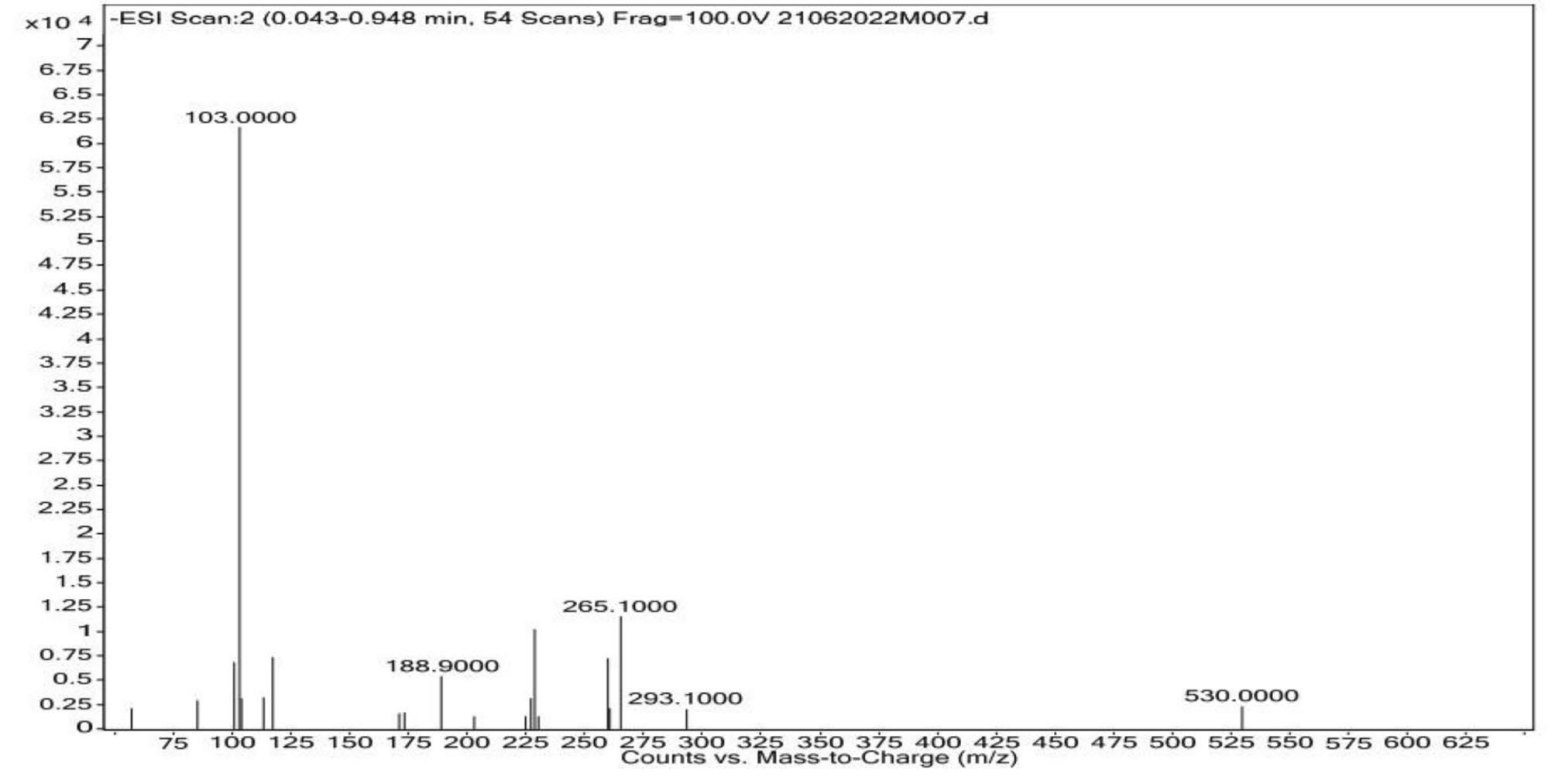
Mass spectrograph of PHB produced by *B. paraconglomeratum*

The mass spectrum revealed the location of the highest mass peak. An ion’s relative abundance is plotted against its m/z value (Fig. 5). The strongest peak in the spectrum is the base peak, which is associated with the relative molecular mass (Mr) of the compound and the peak is known as the molecular peak [M]•+. The carbonyl and hydroxyl groups of the related hydroxyalkanoates were identified using the specific molecular peaks. In the PHB of *B. paraconglomeratum*, the hydroxyl end of methyl 3-hydroxybutyric acid was depicted by the 103.0000 m/z peak in the mass spectrum. The compound analysis and molecular formula for PHB matches the molecular ion peak in the mass spectrum at 103 m/z quite well.

### Thermal analysis

**Fig. 6:**
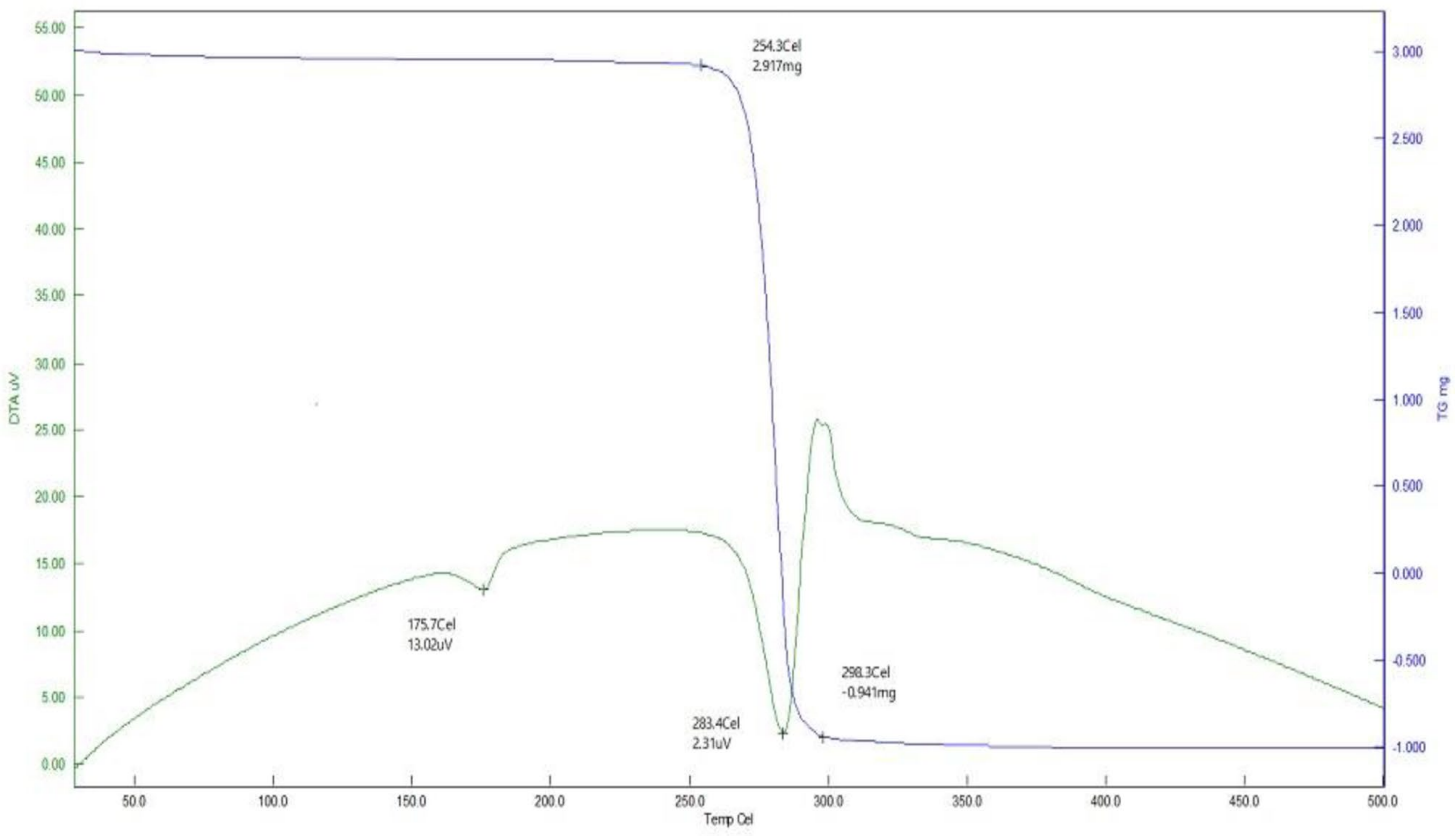
Thermal analysis of *B. paraconglomeratum’s* PHB by TGA

**Fig. 7:**
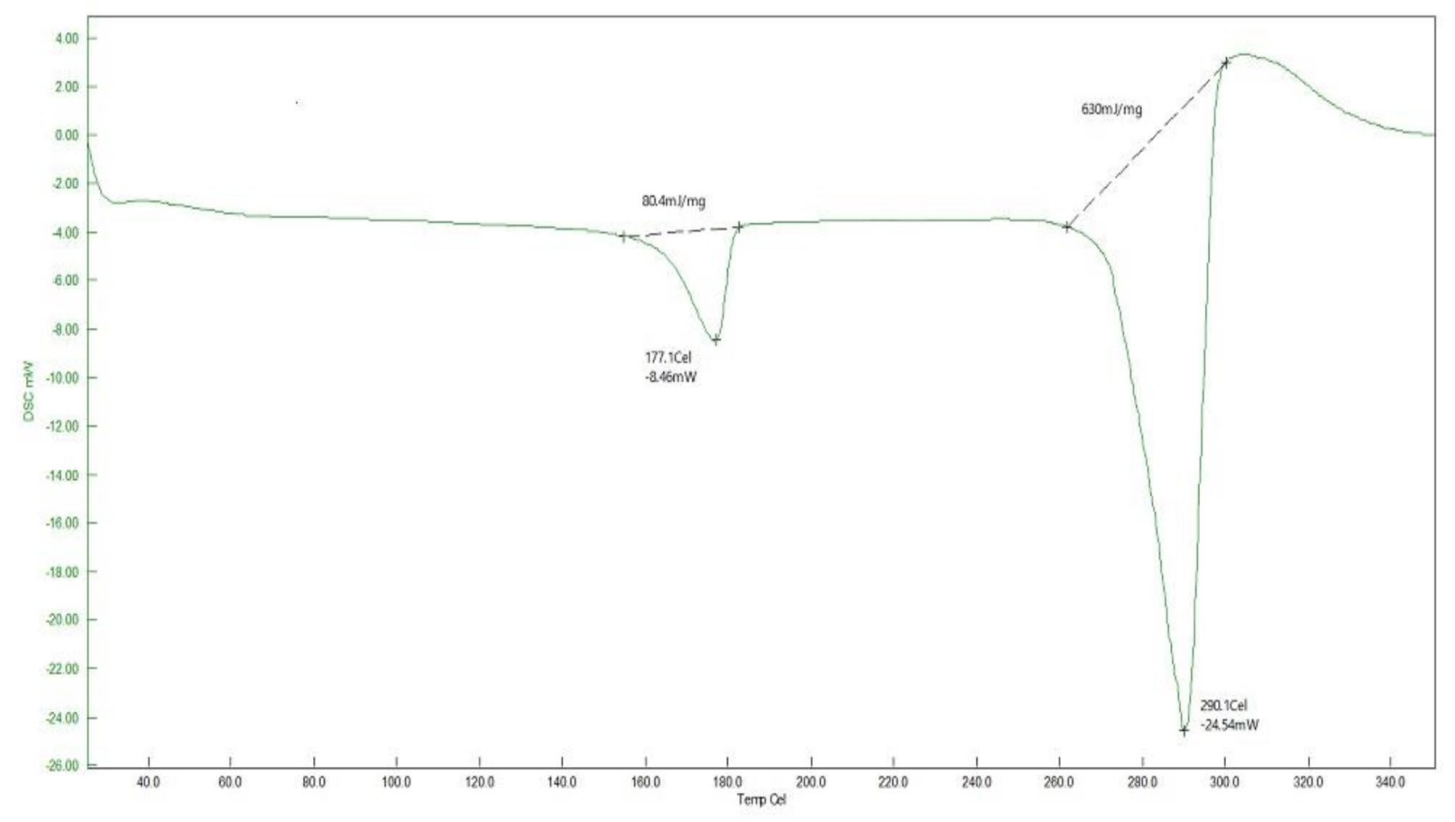
DSC analysis of PHB produced by *B.paraconglomeratum*

Thermal study suggests that PHB produced by *B. paraconglomeratum* has higher thermal stability or greater resistant to thermal degradation. The TGA curve (Fig. 6) showed a glass transition temperature at 175.7 °C, and the obtained polymer in the extract began to lose its mass from 240.2 °C and decomposed completely around 283.4 °C. From the DSC curve (Fig. 7) the melting temperature of the PHB in the extract was approximately 177.1 °C and it was completely decomposed at 290.1 °C. Overall, the mass of PHB was decreased as temperature increased.

### Viscosity and mechanical properties

The viscosity was measured. In general, this was used to identify viscous biopolymers in the samples. In comparison with the standard PHB, the air-dried PHB also has good mechanical qualities, as shown in Table 2.

**Table 2:** Mechanical and thermal analysis of PHB films of *B.paraconglomeratum*

A.	Mechanical Properties	Air-dried PHB Film	Standard PHB
1.	Tensile strength (MPa)	24.2	33.5
2.	Young’s modulus (MPa)	1893	2100
3.	Viscosity (mPa.S )	1.0153	1.1570
4	Elongation at break %	7.65%	8.57%
**B.**	**Thermal analysis**		
4.	Melting temperature –Tm (°C)	177.1	180.7
5.	Transition temperature -Tg(°C)	175.7	223.1
6.	Decomposition temperature –Td(°C)	283.4	290.1

*MPa - Megapascals (unit of pressure), (°C)-Degree Celsius

## Discussion

*Brachybacterium paraconglomeratum* MTCC 13074, a halophilic bacterium is an excellent candidate for PHB production because it has all the ideal features like highly adaptable, non-phathogenic, and having low contamination risks. The isolate has produced a substantial amount of PHB same as recombinant *E. coli* [18]. The produced PHB was characterized by several analytical methods, to determine the chemical structure of the extracted biopolymer. The obtained FTIR spectra had a strong concordance with those of pure PHB as well as previously published spectral information [19]. According to the studies, the PHB functional group was confirmed to be C = O. In the enhanced production of PHB using *Enterococcus faecium* KT722772, C = O and CH_3_ were confirmed at 1700 and 1300 cm^− 1^ stretches [20]. The IR spectrum was comparable to the observations of PHB produced by the *Lysinibacillus sphaericus* BBKGBS6 [21] *Bacillus subtilis* strain [22] and other Bacillus sps. [4], According to previous reports, the peaks of carbonyl groups in bacterial PHAs such as PHB + PHA copolymers and mcl-PHAs may fluctuate [23].

Gas chromatography is an extremely effective method for quantifying and characterizing PHB’s molecular structure. GC analysis requires the depolymerization of PHB into acids, diols or esters [8]. In this study, the existence of PHB in the extracts was confirmed by GCMS by indicating four derivatives of the biopolymer with the retention times of 21.140, 22.635, 30.096, and 35.441 min respectively. The derivatives like 3-hydroxybutyric acid methyl ester, Tetradecanoic acid, pentadecanoic acid methyl ester, were noticed in the PHBV standard [24]. Usually, the monomer methyl ester of PHB is 3-hydroxybutyric acid methyl ester, while the trimer and tetramer methyl esters of 3-HV and PHB are pentadecanoic acid methyl ester and hexadecanoic acid methyl esters respectively [25]. Mostafa et al. [26] and Sriyapai et al. [27] detected similar kind of methyl ester derivatives in PHBV standard and PHB of *Paraburkholderia sp*. These observations confirmed the obtained polymer from *B. paraconglomeratum* as PHB.

NMR has been used for evaluating multiple aspects of PHB like conformational analysis, cellular content, monomer composition, monomer linkage sequence, copolymer analysis etc. The ^1^H NMR revealed resonances for the side groups of hydroxybutyrate i.e., (CH3) at 1.224 ppm, a singlet for (CH) at 5.163 ppm and a doublet for (CH2) at 2.164, and 2.072 ppm. The ^13^C NMR spectra indicated the peaks at 19.71, 40.75, 67.63 and 169.43 ppm for (CH3), (CH2), (CH) and (C). Compatible resonance reports were obtained from the PHB and PHBV produced from the *Cupriavidus necator* [28]. The composition and chemical shifts of hydroxybutyrate were reported as identical to the NMR data of *Bacillus aryabhattai* [24], *Bacillus cereus* [29] and yeast cells [19, 30]. The ^1^H and ^13^C NMR results confirmed the presence of polyhydroxybutyrate in the extract. On the mass spectrograph, the 3HB of *B. paraconglomeratum* revealed a molecular peak at 103.0000 m/z. The results are similar to the 103.2285 m/z peak of *Pseudomonas plecoglossicida* [31] and the 103.02 m/z peak of *Bacillus cerus* SH-02 [32].

The thermal behavior of *B. paraconglomeratum* PHB was thoroughly investigated using non-isothermal techniques such as TGA and DSC to ensure its suitability for processing, industrial application, and thermal recycling [6, 14, 33]. The T_m_, T_g_ and T_d_ values relate to the PHB reported by Balakrishna Pillai [24] from *Bacillus sp.* NA10 and Sharma [5] from *P. putida* 46,123. The T_d_ for PHB produced by *B. paraconglomeratum* ranged from 248.6 to 258.3 °C and the T_m_ ranged between 138 and 166 °C which was considerably higher than that of commercially synthesized biopolymers [34]. The same was observed with the PHBs of *Paracoccus homiensis* [35].

On the other hand, a viscosity of about 1.0153 mPa was obtained by *B. paraconglomeratum* PHB. Sustainable viscosity values would arise under optimized conditions [15]. The polymers made from octanoic, hexanoic, nonanoic, and biodiesel fatty acids displayed reduced complex rheology as angular frequency increased. This decline is related to low melting stability. An increase in viscosity may indicate an increase in the concentration of PHB in the samples, which can be considered a screening method for PHB producing microbes. Interestingly, many studies have explained that the biopolymer’s thermal behavior and mechanical strengths may be greatly enhanced due to the hydroxyvalerate content of the samples [36]. Commercial polymers such as Mirel F1006, Mirel 3002 and P229 have a complex viscosity and the same complexity is exhibited by microbial biopolymers [34]. Significant rheological values were obtained in the study for PHB and airdried films. The PHB films are potential material for a variety of applications due to their versatility and biodegradability, which efforts to lessen the environmental impact of plastic waste in various industries. The properties such as tensile strength, elongation at break and Young’s modulus were noted. The values obtained are compared to the standard PHB and Cynobacterial PHB [37, 38]. The high or low mechanical strengths depend on the molecular weight of the extracted polymer, and the mechanical strength of PHB is positively correlated with its molecular weight [39, 40].

## Conclusions

PHB gathered the interest in bioplastic industry due to environmental concerns and the transition toward more sustainable plastic alternatives. PHB is the material of choice for biomedical applications, packaging, and molded objects since it is completely biodegradable and has sufficient mechanical properties. This will drastically reduce the amount of non-biodegradable waste production. In conclusion, using halophilic bacteria is a viable strategy for finding biotechnological solutions to problems related to the environment and industry. It is crucial to understand the physicochemical, thermal and mechanical characteristics of PHB before using it in applications. The studies with GCMS, FTIR, NMR and mass spectrometry revealed that PHB produced from *B. paraconglomeratum* is a linear polymer made up of repeating units of 3-hydroxybutyrate and the PHB films demonstrated excellent thermal and mechanical stability. As a result, the *Brachybacterium sps* reported in the current study are well utilized in the production of PHB and in the formation of PHB films. Our future objective is to enhance the yield of PHB from *B. paraconglomeratum* MTCC (13074) by using various renewable waste sources, molecular approaches and invivo studies of PHB Films.

## Methods

### Bacteria and production of PHB

*B. paraconglomeratum* was used for PHB production. This strain was isolated from the estuarine habitat and selective staining techniques with Sudan black B and Nile blue A was performed to identify PHB granules. The lyophilized culture was used for the production. The media contains 2% dextrose, ammonium sulfate (10 mM), potassium phosphates (1 M), MgSO_4_ (10 mM), NaCl (1.5%), trisodium citrate 0.5 g/L, pH-7.0 and were cultivated at 35 °C/72 h at 160 rpm. For the extraction, 5 ml of 72-hour culture was used. Initially, the pellets were treated with 2.5 ml of 4% sodium hypochlorite and 2.5 ml of hot chloroform and incubated at 32 °C for 1 h. Three distinct phases were identified after being subjected to 10,000 rpm. The organic (bottom) phase was collected and precipitated by 1:1 ethanol/acetone [17] and the precipitate was subjected to physicochemical characterization.

### Characterization of PHB produced by ***B. paraconglomeratum*** MTCC 13074

#### Fourier transform infrared spectroscopy (FTIR) analysis

The functional groups that are present in the extracted PHB were studied using FTIR (Bruker). Taking 1 ml of liquid extract of PHB, a chloroform solution was directly applied to the window and scans were recorded. The IR spectra recorded the peaks of the samples in the spectral range of 4000–400 cm^− 1^ [41]. Finally, the spectra were examined by comparison to the spectra of standard PHB.

#### Gas chromatography-mass spectrometry (GCMS) analysis

Initially, the sample was subjected to methanolysis. In the present study, the method of methanolysis was followed by Juengert [42]. In a screw-capped glass bottle (20 mL capacity) with a polytetrafluoroethylene cap, 10 mg of extracted PHB, 1 ml chloroform and 1 ml acidified methanol (15% methanol in H2SO4) were mixed and heated in an oil bath at 100 °C for 2 h. After incubation, the bottle was filled with 1 ml chloroform containing an internal standard of 0.2% methyl benzoate v/v and 1 ml deionized water for phase separation. The bottom organic phase was collected and dehydrated with anhydrous Na2SO4 and an aliquot of 1 μl was injected into the Shimadzu GC–MS QP2010S gas chromatograph, which was equipped with an Rxi-5Sil MS (30 m × 0.25 mm × 0.25 μm). Programmed at 1 μl injection volume, an injection temperature of 250 °C and a split mode with a 1:8 split ratio. The recommended flow rate was fixed at 0.7 μl/min. Flame ionization detectors (FIDs) operating at 275 °C are used to detect the phenomenon. The peaks were compared to mass spectral libraries (NIST 17 and Wiley) for the determination of chemical species [43].

#### Nuclear magnetic resonance (NMR) spectroscopy

An Ascend™ 400 (Bruker) spectrophotometer was used to perform NMR analysis of the PHB sample. Purified samples were used to record ^1^H and ^13^C NMR spectra. After 5 mg of the material was dissolved in deuterated CDCl_3_, the solution was analyzed at 400 MHz. A temperature of 25 °C and a pulse repetition time of 3 s were used to record the spectrum. The enhanced proton resonance in ^1^H NMR and the carbon resonance obtained in ^13^C NMR were compared to the peaks of a standard PHB resonance [12].

#### Mass spectrometer analysis

The prepared polymeric composition of the extracted polymer was sampled into the Agilent 6430 triple quad LC-MS/MS for the analysis of the monomeric composition and to estimate the molecular composition [44]. Isocratic elution was carried out at a flow rate of 0.003 mL min^− 1^ of the mobile phase (0.001 M chloroform). With mixed type ionization, the gas temperature was set to 325 °C and the vaporizer temperature was set to 250 °C. The sample was injected at a rate of 5.0 μl/s and detected over 11.5 min. The molecules were broken down into charged ions, and ions of varying masses and intensities traveled down the routes and hit the detector. Data was analyzed using the Mass Hunter workstation program [45].

#### Thermal gravimetric analysis (TGA)

TGA was performed on a HITACHI STA7300 (Japan). The instrument temperature was raised at a rate of 10 °C/min to a level that was significantly higher than the breakdown temperature of the polymers (500 °C). The weight of the sample is plotted against the temperature or the time. As a comparison, the obtained TGA plot of the extract was compared to standard PHB [33, 46].

#### Differential scanning calorimetric (DSC) analysis

DSC (HITACHI, Japan) was used to determine the physical characteristics such as the thermal transitions of polymeric materials. In a differential scanning calorimeter, the temperature was increased from room temperature to 350 °C at a rate of 10 °C/min while nitrogen was flowing [33, 47].

### Viscosity

Viscosity was measured using an Antan paar rolling ball viscometer (Germany). This method was developed for the first time to test the viscosity of bacterial PHB. This was evaluated with the ideal operating volume of 5 ml. The spindle/capillary tube was filled with the PHB sample, the given ball was inserted into the spindle and the viscosity was measured at 37 °C [48]. Both kinetic and dynamic viscosities of the PHB are displayed. The kinetic viscosity has been interpreted in the majority of reports [49].

### PHB films

The viscous PHB extract was air-dried in a glass plate to form thin films before being tested using a universal testing machine. Young’s modulus, tensile strength, and elongation at break were measured under ambient circumstances [38].

## Data Availability

The datasets generated during and/or analyzed during the current study are available from the corresponding author upon reasonable request.
